# Social Integration and Risk of Dementia Among Older Adults

**DOI:** 10.1093/geroni/igaa057.1532

**Published:** 2020-12-16

**Authors:** Yan Zhang, Shannon Shen, Yulin Yang

**Affiliations:** 1 Michigan State University, East Lansing, Michigan, United States; 2 Texas A&M University-San Antonio, San Antonio, Texas, United States; 3 University at Buffalo, Buffalo, New York, United States

## Abstract

We examine the relationship between social integration and cognitive impairment and dementia among older adults using longitudinal data from Waves 1-8 of the National Health and Aging Trends Study (NHATS). The sample includes 7,492 respondents age 65 and older at baseline. We test multidimensional measures of social integration and cognitive well-being using discrete-time hazard models. The risk of dementia is calculated by a series of performance-based tests. Measures include levels of dementia: no dementia, cognitive impairment not dementia (CIND), and dementia, and three domains of cognition functioning: orientation, executive function, and memory. Social integration is an additive index measured by several questions, including marital status, living arrangement, social network, social contact, and social participation. Our results indicate that people with higher social integration have a lower risk of both cognitive impairment (not dementia) and dementia compared to those with lower social integration. This pattern continued across specific domains of cognitive functioning, including lower risk of orientation impairment, executive function impairment, and memory impairment for those with higher social integration. Tests of both gender and racial interactions did not yield any significant differences. Our findings demonstrate the strong association between social integration and lower risk of dementia among older adults. This study can speak to policy makers as the life expectancy of Americans increases and the aging population grows, highlighting the importance of giving support to older adults who are lack of social connectedness.

